# CD8 ^+^ T-cell responses in vaccination: reconsidering targets and function in the context of chronic antigen stimulation

**DOI:** 10.12688/f1000research.14115.1

**Published:** 2018-04-27

**Authors:** Gabriela Cosma, Laurence Eisenlohr

**Affiliations:** 1 Department of Microbiology and Immunology, Thomas Jefferson University, Philadelphia, PA, USA; 2 Perelman School of Medicine, University of Pennsylvania, Philadelphia, PA, USA; 3 Department of Pathology and Laboratory Medicine, Children’s Hospital of Philadelphia Research Institute, Philadelphia, PA, USA

**Keywords:** CD8+ T cells, vaccination, cancers, chronic infections

## Abstract

Cytotoxic CD8 T cells play important roles in eliminating infected and transformed cells. Owing to their potential for therapeutic applications, significant efforts are dedicated toward developing CD8 T cell–based vaccines. Thus far, CD8 T-cell vaccination strategies have had limited success therapeutically in contrast to those targeting antibody-based immunity. However, if the current challenges and gaps in the understanding of T-cell biology are overcome, the full potential of rational CD8 T-cell vaccine design might be realized. Here, we review recent progress in this direction, focusing on target selection and maintenance of function in the settings of chronic infections and cancers.

## Introduction

Safe and effective vaccines have significantly reduced and even eradicated many pathogen-related human health threats within the past century ^1^. Cancers and a handful of chronic viral infections remain the last frontier of prevention and treatment through vaccination. Historically, the vast majority of vaccines have focused on inducing humoral immunity, yet the remaining immunological challenges may benefit from the engagement of cell-mediated immunity as well ^2^. In recent decades, large gains have been made in the development of CD8 T cell–based vaccines; however, complete protection in both pre- and post-exposure cases has not yet been achieved. CD8 T-cell vaccine development entails unique challenges that include (a) identifying and validating CD8 T-cell targets that correlate with protection and (b) maintaining and restoring optimal functionality of CD8 T cells in an immunosuppressive or chronic infection environment. This review focuses on CD8 T-cell responses to vaccination and advances in these challenge areas from the past few years and particularly on cancers and HIV chronic infection. Promising directions for vaccine design include the identification of non-canonical CD8 T-cell targets and finding means of sustaining CD8 T-cell function through the combination of immunotherapy and inhibitor receptor blockade.

## CD8 T-cell target recognition and identification

Unlike antibodies, which recognize conformational epitopes exposed on the surfaces of proteins that have achieved tertiary structure, CD8 T cells recognize 8–11 amino acid peptide fragments displayed at the plasma membrane in complex with major histocompatibility complex (MHC) class I molecules ^3, 4^. In humans, MHC class I molecules are encoded at three human leukocyte antigen (HLA) loci (A, B, and C). Peptide fragments arise primarily from standard protein degradation by the multicatalytic proteasome and are further processed by peptidases in the cytosol or the endoplasmic reticulum where MHC loading occurs ^5^. Linear epitopes can be derived from any protein domain, including buried internal regions. This is important since, owing to cellular localization (for example, viral polymerases that are confined to the cytosol), not all pathogen- or cancer-related proteins are accessible to antibodies but instead can be detected by CD8 T cells. These target characteristics of CD8 T-cell epitopes offer a broader range of options for identifying an infected or transformed cell and naturally complement humoral immunity.

Epitope identification continues to be a largely empirical process. The two main methods of identifying T-cell targets are mass spectrometry (MS) analysis and epitope mapping ^6, 7^. The first approach isolates peptides from target cells and identifies them through MS. Thus, actively processed and displayed peptides can be identified directly. Through this technique, post-translationally modified or non-canonical peptides (for example, spliced) can also be recognized ^8^. Nevertheless, MS is resource-intensive and despite technical advances still requires large amounts of starting material along with subsequent validation of identified epitopes in a CD8 T cell–based assay. Despite gains in sensitivity, the technique may not detect peptides displayed at low levels that nevertheless can activate CD8 T cells.

Epitope mapping interrogates T-cell responses to a set of synthetic peptides derived from the primary amino acid sequence of pathogenic genomes ^7, 9^. Two main approaches have been used for designing the peptide library: overlapping peptides or the use of a predictive algorithm. Screening by either approach becomes substantially more complex and cost-prohibitive the larger the genome included in the analysis. Thus, the overlapping-peptide approach is best suited for individual proteins or pathogens with small genomes and can be used with broad applicability to probe T-cell responses across all MHC alleles. On the other hand, use of a predictive algorithm allows for greater coverage of a larger genome, since the library is designed for a unique MHC allele binding pattern, thus restricting the number of peptide candidates. Though rapidly evolving, current MHC binding predictive algorithms remain imperfect and strongly immunogenic epitopes can be missed. Overall, synthetic libraries designed by either method require validation through a T-cell recognition assay—typically enzyme-linked immunospot detection of gamma interferon—since not all of the predicted epitopes are presented or produce an immunogenic response. More importantly, non-linear epitopes or post-translational modifications cannot be accounted for by this process and can be entirely unrepresented in conventional searches for T-cell epitopes. Finally, only some of the epitopes identified and validated through either method will turn out to be protective and further testing will be required to assess their utility in context ^10^.

## New sources of cancer immunotherapeutic peptides

CD8 T-cell vaccine design for cancers requires the identification of tumor-specific epitopes and stimulation of robust and effective CD8 T-cell responses against the epitope. Since tumors are derived from a body’s own cells, many epitopes will arise from self-antigens through over-expression of proteins in transformed cancer cells or display of antigens normally present in immunopriviledged sites ^11^. These antigens are typically weak immunogens because of central and peripheral tolerance mechanisms acting on the T-cell repertoire. However, tumor cells can also give rise to neo-antigens through somatic mutations resulting in modified amino acid sequence or through aberrant post-translational modifications (for example, hyperphosphorylation) ^11, 12^. Epitopes derived from neo-antigens have a much greater potential for eliciting immunogenic responses since they are not subject to central tolerance ^11^. Although cancer-induced immunosuppression often stunts T-cell responses against the tumor, inhibitory receptor therapy can help to restore T-cell function, as discussed later in this review.

Large but still untapped sources of potentially immunogenic neo-antigens are spliced peptides. The proteasome cleaves proteins into linear peptide fragments; however, in 2004, it was first reported that the proteasome can also splice together short peptide fragments, typically from the same protein, resulting in the formation of discontinuous epitopes ^13^. The efficiency of the mechanism through which this transpeptidation reaction takes place is not well understood ^14– 20^; however, new data suggest that spliced peptides are generated with the same efficiency as linear epitopes ^21^. At the same time, the frequency of spliced peptides has been considered extremely rare, and only six cancer-related, spliced peptides have been identified in the past decade ^22^. However, using cutting-edge bioinformatics and liquid chromatography–MS methods, a recent study determined that a large fraction of class I ligands are, in fact, spliced proteasomal products ^23^. By this new account, up to one third of protein diversity is represented by spliced peptides, and these spliced products account for one quarter of the total presented peptides, uncovering a potentially large pool of untapped immunogenic peptides that could be beneficial for targeting cancer cells ^23^. In addition, the first bacterial account of CD8 T-cell responses to spliced epitopes has been reported in a *Listeria monocytogenes* infection, expanding the applicability of spliced peptides to infectious agents ^24^. The authors used a combination approach of *in vivo* and *in silico* predictive algorithm methods to identify spliced peptides, creating a workflow that could be employed in the future for spliced epitope identification. An important area to investigate is the extent of splicing in transformed cells compared with normal cells since the first spliced epitopes discovered through low-throughput methods are all tumor antigens. Lastly, since spliced epitopes are more frequent than anticipated, vaccination platforms should take into consideration the use of whole antigens and maintenance of processing requirements such as access to the proteasome.

## Non-canonical presentation of HIV epitopes

HIV is a formidable challenge for many reasons. Owing to the high variability and extensive glycosylation of the HIV envelope protein, antibodies have been largely ineffectual in preventing viral entry. CD8 T cells have been reported to help control viral replication; however, high mutation rates allow for viral escape. Another factor is T-cell exhaustion, a hallmark of chronic infections, which is characterized by increased expression of inhibitory receptors, loss of T-cell functions such as cytokine production and cytotoxicity, and transcriptional and metabolic changes. Lastly, no identified epitope presented through canonical MHC I presentation (on HLA-A, -B, and -C class I molecules) has demonstrated complete protection from HIV.

Recent discoveries from the Picker lab point to non-canonical epitopes presented on HLA-E molecules as a means of eliciting immunogenicity and protection ^25^. HLA-E typically presents a unique set of peptides, derived from the leader sequences of classic MHC molecules. Their primary function is thought to be the interaction with natural killer cells and their inhibitory receptors NKG2 A-C in order to prevent cell lysis. Previously, only a handful of pathogenic infections were shown to produce CD8 T cells specific for HLA-E molecules loaded with peptides that mimic leader sequences. Surprisingly, in 2016, HLA-E molecules were reported to present simian immune deficiency virus (SIV) peptides and elicit CD8 ^+^ T-cell responses depending on the virus immunization platform ^25^. This phenotype is specifically induced by a cytomegalovirus (CMV)-based vector platform, which upregulates expression of the HLA-E molecule; in contrast, pox vectors induced presentation of classic MHC I molecules ^26^. HLA-E–restricted CD8 T cells appear to carry out the same functions in terms of cytotoxicity and cytokine release as those that are classically induced through MHC I presentation. Furthermore, CMV vaccine vectors, now known to induce primarily HLA-E–restricted CD8 T cells, have been previously shown to control and clear SIV infection in 50% of vaccinated rhesus macaques ^27^. The effects of CMV vaccine platforms on CD8 T-cell development are reviewed in greater depth here ^28^.

The use of HLA-E–restricted CD8 T cells is an appealing approach because of naturally low polymorphism in the human population and the potential for use of highly relevant, established animal models. Hundreds of classic HLA A/B/C alleles exist in the human population; of these, only the HLA-B27 has been found to be associated with better virus replication control in HIV ^29^ and also hepatitis C virus ^30^. HLA-B27 is present in only 6.1% of humans ^31^. In contrast, HLA-E is expressed in only two allelic forms, characterized by a single amino acid substitution ^32^, and this difference has no impact on peptide binding. In fact, the peptide binding groove is conserved between humans and all species of macaques ^33^. Furthermore, a 2017 study confirmed the conservation of HLA-E expression levels, T-cell response patterns, and the ability to present identical peptides between humans, rhesus macaques, and cynomolgus macaques, which supports the establishment of physiologically relevant animal models for HIV and SIV vaccine platforms ^34^. The applicability of this approach is now being expanded to other pathogens since HLA-E–presented epitopes have been recently discovered in a tuberculosis infection setting through the use of in-depth MS ^26^. One particular epitope elicited immunodominant CD8 T-cell recall responses in 14 out of 16 donors, further suggesting the broad applicability of HLA-E–restricted T cells ^26^. Many gaps remain in understanding how antigen presentation is shifted from the classic MHC molecules toward HLA-E through CMV vector vaccination and whether HLA-E restriction or peptide specificity provides the protective advantage in an SIV setting. Nevertheless, HLA-E–restricted CD8 T-cell responses are an exciting new direction because of their broad applicability to a large patient population as well as demonstrated protective capabilities.

## Restoring and maintaining CD8 T-cell function

The presence of tumor-infiltrating lymphocytes, including effector CD8 T cells, has been associated with better prognosis in a variety of cancers. However, CD8 T-cell function is frequently suppressed by the tumor microenvironment. A recently established strategy for restoring T-cell function, especially in cancer treatment, is checkpoint inhibitor blockade therapy ^35^. Inhibitory receptors, most prominently PD-1 and CTLA4, are expressed on the CD8 T-cell surface upon activation and interact with ligands expressed by antigen-presenting cells ^36^. While the main role of inhibitory receptors appears to be the dampening of immune responses in order to prevent immunopathology, in a prolonged antigen exposure scenario, this ultimately leads to CD8 T-cell dysfunction. So far, inhibitor blockade therapy has produced remarkable results in clinical trials, although only about 20% of patients respond to treatment ^36^. Even in responding patients, restoration of CD8 T-cell function is not permanent and continuous immunotherapy is necessary ^36^. Thus, an essential question regarding T-cell dysfunction in cancer is whether the process develops in the early stages of tumor formation or whether it progresses gradually along with cancer metastasis and further exposure to antigen. Understanding the timing and molecular mechanisms of T-cell dysfunction can redirect the development of CD8 T-cell vaccination strategies in conjunction with checkpoint inhibitor therapy blockade.

A recent study using a mouse model reports that T-cell dysfunction is initiated during the pre-malignancy phase, prior to tumor formation and subsequent induction of an immunosuppressive environment ^37^. Thus, T-cell dysfunction is established early in tumorigenesis and appears to be driven by persistent antigen stimulation, analogous to the exhaustion scenario in a chronic infection setting ^37^. Inhibitor blockade was found to partially alter the gene expression of tumor-specific dysfunctional T cells but did not permanently re-shape the underlying epigenetic landscape ^38^. In support of this finding, a subsequent study characterized the changes in CD8 T-cell chromatin accessibility that take place early in tumorigenesis ^39^. Two distinct developmental phases were identified. Initially, epigenetic changes are plastic and the CD8 T cells are amenable to reversal of the dysfunction phenotype within the first week of tumor challenge with inhibitor blockade treatment. However, prolonged antigen exposure led to a fixed chromatin landscape characterized by permanent dysfunction within a month of tumor implantation. PD-1 high tumor-infiltrating lymphocytes from human melanoma and non-small cell lung cancer biopsies were found to contain a mixture of reversible and irreversible chromatin states. This suggests that a small population of CD8 T cells might still be amenable to functional restoration and could provide a basis for identifying patients who would be responsive to inhibitor blockade therapy. It will become increasingly important to understand the underlying differences between the two chromatin states and how to prevent transition to the fixed epigenetic state for induction of *de novo* responses when formulating CD8 T-cell vaccines. Recent clinical trials have begun exploring combination therapy of inhibitor blockade along with neo-antigen–based CD8 T-cell vaccination. Since naïve T cells still have the potential to develop into functional effector and memory cells, this strategy may yield the best results yet. It will be interesting to learn whether this combination prevents the development of dysfunction in the CD8 T-cell compartment.

T-cell dysfunction characterized by inhibitor receptor expression is a common feature in both cancer and chronic infections. However, inhibitory receptor blockade therapy is used much less prominently in the clinical setting of viral infections (for example, HIV), even though these receptors have been studied intensively in mouse models of T-cell exhaustion. Recent reports suggest that, as with cancer, the exhaustion phenotype originates in the early stages of the initial infection. In a mouse model of chronic lymphocytic choriomeningitis virus (LCMV) infection, PD-1 blockade temporarily restored the function of exhausted T cells but failed to induce long-term functional memory cells in the absence of treatment ^40^. Exhausted T cells were found to contain an inflexible epigenetic profile that was not altered by PD-1 blockade. In addition, methylation of the PD-1 promoter in CD8 T cells, which promotes expression of the inhibitory receptor, was found to occur early in the infection time line and to be maintained in the long term ^41, 42^. Moreover, PD-1 promoter methylation was shown to persist even in the absence of antigen ^43^. Much like in a cancer setting, inhibition of PD-1-PD-L1/2 interactions does not reverse the epigenetic changes ^44, 45^ and continuous therapy is likely necessary in order to maintain T-cell function. However, restoration of the CD8 T-cell compartment with functional cells may still be possible. In several murine studies, a subset of stem-like CXCR5 ^+^ memory follicular T cells, which retains a de-methylated PD-1 promoter, was found to account for the proliferative burst and repopulation of functional CD8 T cells after inhibitor blockade therapy ^45– 48^. Moreover, inhibition of *de novo* DNA methylation in naïve CD8 T cells through a conditional knockout mouse model results in proper development and functionality of the T-cell compartment, even under chronic antigen stimulation conditions ^44^. *De novo* T-cell priming in conjunction with methylation inhibitors could be another avenue for regenerating the CD8 T-cell compartment in a chronic infection setting provided that specific, low-toxicity means of inhibiting DNA methylation can be identified. More detailed analyses of epigenetic changes affecting checkpoint inhibitor genes have recently been summarized elsewhere ^36, 49, 50^. Finally, earlier findings about the synergistic effect of administering combination therapy of PD-1 inhibitors along with the interleukin-2 (IL-2) cytokine, which improves the outcome of chronic LCMV infection over inhibitory therapy alone, might be re-examined from an epigenetic viewpoint. This combination therapy produced a large expansion of antigen-specific CD8 T-cells, further characterized by decreased surface expression of PD-1 and an increase in the memory T-cell markers IL-7Rα and the transcription factor T-bet and, resulted in a higher responsiveness to PD-1 blockade treatment ^51^. It would be interesting to examine the methylation state of the PD-1 promoter in these CD8 T-cells in order to determine whether the epigenetic landscape associated with exhaustion has been reversed.

## Concluding remarks

CD8 T-cell vaccine development against cancers and chronic infections have common challenges. In both cases, although antigen-specific CD8 T cells can be elicited through vaccination, this does not translate into long-term protection. To overcome this, both re-evaluation of T-cell vaccine targets and devising methods for the maintenance of functionality are likely necessary. A promising trend redirects attention to non-conventional CD8 T-cell epitopes, such as spliced non-linear peptides, and the non-canonical antigen presentation molecule HLA-E. Improved target selection can be further supported by vaccination platforms that include checkpoint inhibitor blockade therapy since maintenance of CD8 T-cell function in prolonged disease states is a fundamental problem. Lastly, a better understanding of the mechanisms behind T-cell dysfunction and checkpoint inhibitors should lead to effective treatment in a wider segment of the cancer patient population but also in chronic infection settings.
